# Insights About the Neuroplasticity State on the Effect of Intramuscular Electrical Stimulation in Pain and Disability Associated With Chronic Myofascial Pain Syndrome (MPS): A Double-Blind, Randomized, Sham-Controlled Trial

**DOI:** 10.3389/fnhum.2018.00388

**Published:** 2018-10-16

**Authors:** Leonardo Botelho, Letícia Angoleri, Maxciel Zortea, Alicia Deitos, Aline Brietzke, Iraci L. S. Torres, Felipe Fregni, Wolnei Caumo

**Affiliations:** ^1^Post-Graduate Program in Medical Sciences, School of Medicine, Universidade Federal do Rio Grande do Sul, Porto Alegre, Brazil; ^2^Laboratory of Pain and Neuromodulation, Hospital de Clínicas de Porto Alegre, Porto Alegre, Brazil; ^3^Anesthesia and Perioperative Pain Medicine, Hospital de Clínicas de Porto Alegre, Porto Alegre, Brazil; ^4^Pain and Palliative Care Service at Hospital de Clínicas de Porto Alegre, Universidade Federal do Rio Grande do Sul, Porto Alegre, Brazil; ^5^Department of Pharmacology, Institute of Basic Health Sciences, Universidade Federal do Rio Grande do Sul, Porto Alegre, Brazil; ^6^Spaulding Center of Neuromodulation, Department of Physical Medicine and Rehabilitation, Harvard Medical School, Boston, MA, United States; ^7^Department of Surgery, School of Medicine, Universidade Federal do Rio Grande do Sul, Porto Alegre, Brazil

**Keywords:** MPS, EIMS, TMS, clinical trial, BNDF, QST

## Abstract

**Background:** There is limited evidence concerning the effect of intramuscular electrical stimulation (EIMS) on the neural mechanisms of pain and disability associated with chronic Myofascial Pain Syndrome (MPS).

**Objectives:** To provide new insights into the EIMS long-term effect on pain and disability related to chronic MPS (primary outcomes). To assess if the neuroplasticity state at baseline could predict the long-term impact of EIMS on disability due to MPS we examined the relationship between the serum brain-derived-neurotrophic-factor (BDNF) and by motor evoked potential (MEP). Also, we evaluated if the EIMS could improve the descending pain modulatory system (DPMS) and the cortical excitability measured by transcranial magnetic stimulation (TMS) parameters.

**Methods:** We included 24 right-handed female with chronic MPS, 19–65 years old. They were randomically allocated to receive ten sessions of EIMS, 2 Hz at the cervical paraspinal region or a sham intervention (*n* = 12).

**Results:** A mixed model analysis of variance revealed that EIMS decreased daily pain scores by -73.02% [95% confidence interval (CI) = -95.28 to -52.30] and disability due to pain -43.19 (95%CI, -57.23 to -29.39) at 3 months of follow up. The relative risk for using analgesics was 2.95 (95% CI, 1.36 to 6.30) in the sham group. In the EIMS and sham, the change on the Numerical Pain Scale (NPS0-10) throughout CPM-task was -2.04 (0.79) vs. -0.94 (1.18), respectively, (*P* = 0.01). EIMS reduced the MEP -28.79 (-53.44 to -4.15), while improved DPMS and intracortical inhibition. The MEP amplitude before treatment [(Beta = -0.61, (-0.58 to -0.26)] and a more significant change from pre- to post-treatment on serum BDNF) (Beta = 0.67; CI95% = 0.07 to 1.26) were predictors to EIMS effect on pain and disability due to pain.

**Conclusion:** These findings suggest that a bottom–up effect induced by the EIMS reduced the analgesic use, improved pain, and disability due to chronic MPS. This effect might be mediated by an enhancing of corticospinal inhibition as seen by an increase in IC and a decrease in MEP amplitude. Likewise, the MEP amplitude before treatment and the changes induced by the EIMS in the serum BDNF predicted it’s long-term clinical impact on pain and disability due MPS.

**The trial is recorded in ClinicalTrials.gov**: NCT02381171.

## Introduction

Myofascial pain syndrome (MPS) encompasses muscle and musculotendinous pain secondary to the development of myofascial trigger points (MTrPS). It is the principal source of pain in about 30% of individuals with musculoskeletal dysfunction, and its primary components are MTrPS, tender points, and taut bands. The trigger point induced central sensitization explains the referred pain and hyperalgesia phenomenon (Gerwin, 2014). Central sensitization (CS) represents an intensification in the activity of circuits and neurons in nociceptive pathways caused by an enhancement in membrane and synapses excitability. In patients with central sensitization, any sensory experience presents with higher amplitude, duration and spatial extent, which reflects a reduced excitatory-inhibitory balance (Schwenkreis et al., 2011; Botelho et al., 2016). Also, the reorganization of the cortex leads to an aberrant and extreme enhancement of pain (Nurmikko et al., 2016).

The transcranial magnetic stimulation (TMS) measures have proven to be useful to assess cortical physiological processes (e.g., inhibition, excitation). Previously, we found that hyperexcitability in the cortical-spinal pathway as measured by motor evoked potential (MEP) amplitude was positively correlated with the disengagement of the descending pain modulatory system (DPMS) (Botelho et al., 2016). Also, in other studies we showed that an imbalance between inhibitory and excitatory systems as indexed by a decreased cortical silent period (CSP) and an enhancement in the intracortical facilitation (ICF) were correlated with a higher levels of pain catastrophizing (Volz et al., 2013), higher trait anxiety and higher rate of disability due to pain (Vidor et al., 2014). Indeed, these set of results indicate that the primary motor cortex (M1) has turned into a target for assessing either neuroplasticity of the cortical-spinal pathway or, as well the cortical reorganization (Schwenkreis et al., 2011; Leite et al., 2017).

These changes in excitatory and inhibitory transmitter systems are confluent with a reduction in the descending inhibitory pathways (Botelho et al., 2016; Thibaut et al., 2017; Thapa et al., 2018). As aforementioned, the dysfunction in the inhibitory corticospinal system involves several different mechanisms, such as the strength of the glutamatergic synapses or the weakening of synapses of the GABA-ergic system. Actually, in this processes of sensitization, the brain-derived neurotrophic factor (BDNF) secreted by astrocytes and glial cells invert the influxes of the chlorite in GABA-ergic neurons and the GABA-ergic system paradoxically increase the excitability (Binder and Scharfman, 2004). Also, the BDNF sensitizes nociceptive neurons in the spinal cord, and it facilitates the activation of *N*-methyl-D-aspartate (NMDA) (Binder and Scharfman, 2004; Coull et al., 2005). Additionally, cumulative data indicate that BDNF effects are likely to be region-specific due to the fact that in the spinal cord it up-regulates pain pathways while in the hippocampus it down-regulates synaptogenesis and neurogenesis (Duric and McCarson, 2007). Notably, according to the previous study, the electroacupuncture increased the serum BDNF around threefold compared to sham (Chassot et al., 2015). However, there is still a gap to advance in the comprehension of the BDNF role in the motor cortex excitability on chronic pain and how can it influence the results of therapeutic approaches, such as the electric intramuscular stimulation (EIMS).

The EIMS is a technique of electroacupuncture used to modulate pain processing in a bottom–up fashion. Although the neuroplasticity processes involved in its effect is not yet well comprehended (Couto et al., 2014; da Graca-Tarragó et al., 2015; Leitch et al., 2017), it has been used to treat some chronic pain conditions, as in MPS and chronic tensional headache) (Couto et al., 2014; Chassot et al., 2015). Among the possible mechanisms involved in its long-term effects points out by the change in the neuroplastic state as assessed by the serum BDNF, the reduced ICF and increased the CSP (Tarragó et al., 2016). However, to date have limited evidence to comprehend the EIMS effect in an integrative assessment including neurophysiological measures that evaluate its impact on the ratio of the inhibitory/excitatory system at the cortical level, on the descending pain modulating system function and the BDNF. Thus, we need novel insights into the EIMS effect into the neural pathways involved in the pathophysiology of chronic MPS.

We investigated the effect of 10 sessions of EIMS to test the primary hypothesis: To provide new insights into the EIMS long-term effect on pain and disability related to chronic MPS (primary outcomes). Also, we tested another secondary hypothesis: (i) if the neuroplasticity state at baseline could predict the long-term impact of EIMS on disability due to MPS we examined the relationship between the serum brain-derived-neurotrophic-factor (BDNF) and by MEP. (ii) If the EIMS could improve the DPMS assessed by the change in the score on Numerical Pain Scale (NPS0-10) at the Conditioned Pain Modulation test (CPM-task) induced by cold water (0–1°C). (iii) And if it changes the cortical excitability parameters indexed by TMS parameters [MEP, ICF, CSP and short intracortical inhibition (SICI)]. We hypothesize that the EIMS analgesia is related to changes at the pain pathways at cortical and infra-cortical levels and that TMS parameters and serum BDNF are reliable markers of neuroplasticity state.

## Materials and Methods

**FIGURE 1:**
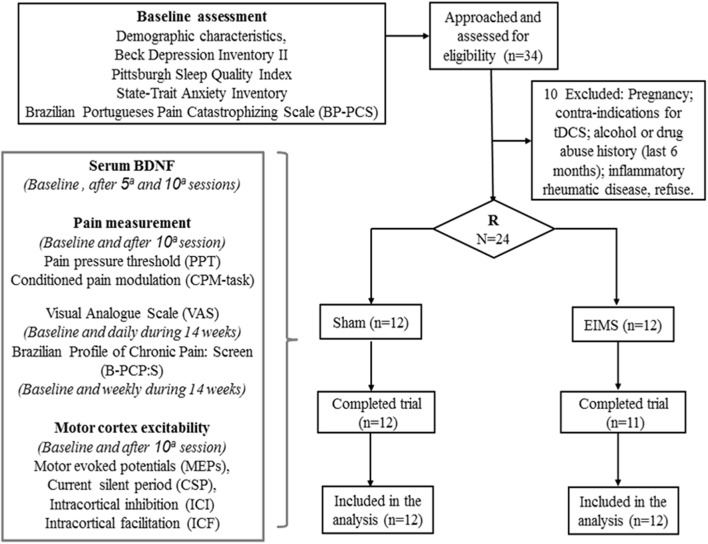
Flow chart showing participants recruitment and progress through the study.

The methods and results are presented according to the CONSORT guidelines (Schulz et al., 2010). The flow chart of the study is displayed in **Figure 1**.

### Design Overview, Settings, and Participants

The Research Ethics Committee approved the protocol of this study at the Hospital de Clínicas de Porto Alegre (HCPA) (Institutional Review Board IRB 100276) following the Declaration of Helsinki. Before participating in this randomized, double-blind, two-group parallel, clinical trial, all patients provided their oral and written informed consent.

Twenty-four right-handed female patients, aged 19–65 years with a diagnosis of MPS in the upper body part were recruited. They should have clinical criteria to MPS (i.e., restricted findings of regional pain, palpable nodules, taut bands, stiffness in the target muscles, as well as the existence of trigger points and tender points). They should report some restrictions for the routine activities due to pain for at least 3 months as assessed using a questionnaire with six specific questions (yes/no). These questions were intended to evaluate interference with personal relationships, work, personal goals, pleasure with activities, and clear thinking (i.e., concentrating, problem-solving, or remembering). To be enrolled, subjects had to give an affirmative answer to one or more of the six questions mentioned above. Moreover, a second independent investigator with more than 10 years of experience to care for patients with chronic MPS confirmed the diagnosis after a standard clinical examination. They should describe the pain as dull, hollow or deep and aggravated during stress. The Neuropathic Pain Diagnostic Questionnaire (DN4) was employed to distinguish neuropathic pain from continuing nociception. To standardize the diagnosis of MPS concerning the severity, only patients with a neuropathic pain component were included (i.e., score equal to or higher than four) on DN4 (Bouhassira et al., 2005).

The exclusion criteria comprised the presence any other pain diagnosis, such as radiculopathy, rheumatoid arthritis, fibromyalgia; previous surgery on the affected areas; constant usage of anti-inflammatory steroids (because they could interfere in TMS measures). Also, patients with oncologic or neurologic disease history, hepatic or kidney insufficiency, and ischemic heart disease or those with criteria to contra-indicated TMS use according to the international guideline were excluded. If they had a history of alcohol or substance abuse during the previous 6 months were also excluded.

### Sample Size Rationale

The sample size was estimated based on previous clinical trial (Dao et al., 1991). A sample size of 22 patients divided into two groups of 11 would permit to detect a difference of 1.5 cm in the pain severity reported on the visual analog scale (VAS) of 10 cm, by a standard deviation (*SD* = 1.2), for a error type I equal to 1% and an error type II equal to 20%, respectively. We included 24 patients (12 per groups) to account for possible dropouts.

### Randomization

We used software to generate the sequence of randomization with a fixed block size of 6. Twenty-four patients were randomly allocated to receive treatment (EIMS or sham). Before the recruitment phase, opaque brown envelopes containing the protocol were prepared. The envelope contained a numerical code corresponding to the allocated treatment. Each envelope was opened in sequence according to the number that existed externally after the participant agrees to engage in the study. Only the physician responsible for administering the interventions was not blinded to treatment.

### Blinding

To guarantee the blinding, during the whole timeline protocol, two investigators who not involved in the patient’s evaluations were responsible for handling the randomization code. Also, to reduce the potential to bias the EIMS was administered by the same physician (with more than 10 years of practice in acupuncture). Furthermore, the participants were requested to debate treatment details only with the physician who administered the EIMS sessions. Pain assessments, psychological tests and measures of cortical excitability by TMS was done by two trained examiners who were blinded to the interventions group.

### Interventions

#### Active Electrical Intramuscular Stimulation (EIMS)

**FIGURE 2:**
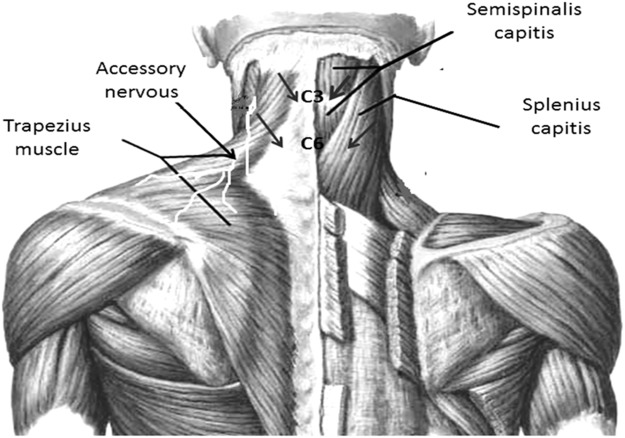
Paraspinal intramuscular stimulation using acupuncture needles. Distance from the spinous process line is 1.5 cm at C3–C4 (splenius capitis muscle); C5–C6 (semispinalis capitis). Accessory nerves were stimulated in front of the anterior border of the sternocleidomastoid by recording the motor responses from the trapezius muscle. Nerve roots involved in the splenius capitis (C3 and C4).

The EIMS was applied using acupuncture needles with guide tubes, 30 mm in length and 0.25 min diameter (Suzhou Huanqiu Acupuncture Medical Appliance Co. Ltd., 218, China) connected to an electroacupuncture device (Sikuro, São Paulo, Brazil). The current was delivered to EIMS in the paraspinal region related to the nerve roots involved in the splenius capitis (C3 and C4) (**Figure 2**) and semispinalis capitis (C2 and C3). The needles were inserted at the paraspinal region at 1.5 cm from the spinous process line (Couto et al., 2014) at the anterior border of the sternocleidomastoid muscle (**Figure 2**). The accessory nerves were stimulated to record a motor response from the trapezius muscle (Fahrer et al., 1974). They received 10 treatment sessions during 20-min at a frequency of 2 Hz (da Graca-Tarragó et al., 2015).

#### Sham of Intramuscular Electrical Stimulation

The same electroacupuncture device (Sikuro, São Paulo, Brazil), described above was used for the sham control condition. However, the output jack plug was broken to avoid no electrical current could move to the patient. We fixed the electrodes at the same spots where active stimulation would be applied. That is, at the paraspinal region at 1.5 cm from the spinous process line, at the border of the sternocleidomastoid muscle and to stimulate the accessory nerves we fixed the electrodes at the anterior border of the trapezius muscle (**Figure 2**). While the stimulator was left in front of the participant for 20 min, to ensure that the flashing diode corresponding to the electrical stimulus was both audible and visible. All patients received ten sessions with 20-min of duration.

### Instruments and Assessments

#### Outcomes

The primary outcomes comprise disability due to pain in the Brazilian Profile of Chronic Pain: Screen (B-PCP:S) and the pain reported on the VAS registered in a diary. Secondary outcomes were the analgesics doses used weekly during the treatment period, the change on Numerical Pain Scale (NPS0-10) during the conditioned pain modulation (CPM-task) and heat pain threshold (HPT). Also, we assessed the cortical excitability measures (MEP, ICF, CSP, and SICI), and daily sleep quality. Below, we described the outcomes assessment in details.

##### Primary outcomes

(a) Pain intensity was evaluated using a 10-cm VAS. The scores in VAS ranged from zero (no pain) to 10 (worst possible pain). We request to patients to report the pain score in the most part of the time in the last 24 h? So, to increase the compliance, an examiner checked the patients’ pain diary during the 12 weeks of follow-up.

(b) To assess the multidimensional pain experience we used the Brazilian Profile of Chronic Pain: Screen (B-PCP:S) (Caumo et al., 2013). The B-PCP:S comprehend three subscales: (i) the subscale to assess the pain severity comprise four items with a possible score extending from 0 to 32). (ii) Subscale to evaluate the pain interference in the routine activities by 6 issues and its score extending from 0 to 36). (iii) Subscale to assessed the emotional burden due to pain by five items and its rating extending from 0 to 25. It was employed at baseline, at the end of the intervention course, and at 2, 4, 6, 8, and 12 weeks after the end of the intervention. The disability related to pain is characterized to be associated with chronic or recurrent discomfort or pain causing restriction (Caumo et al., 2013). Thus, we considered that higher scores on the B-PCP:S indicated higher disability or functionality at home, at work, during social circumstances and when experiencing more significant amounts of the emotional burden.

##### Secondary outcomes

(c) Supplementary analgesia use: Patients could use additional analgesic drugs (acetaminophen, ibuprofen, Dorflex^®^, tramadol or codeine) for pain relief, if necessary. They were authorized used 750 mg of acetaminophen up to four times per day (QID) and 200 mg of ibuprofen up to QID as rescue analgesics. If these drugs were not sufficient, patients could use Dorflex^®^ (Sanofi Aventis, São Paulo, Brazil; 35 mg orphenadrine citrate combined with 300 mg dipyrone and 50 mg caffeine). If they persisted with pain, It was allowed to use 60 mg of codeine up to QID or tramadol three times per day (TID). Patients recorded the rescue of analgesic used in a pain diary, which was checked during every intervention session and each visit of the follow-up. For analysis, we considered the total analgesic dose used throughout treatment.

(d) The Quantitative Sensory Testing (QST) assessed the HPT. We used a thermode (30 × 30 mm) in a computer Peltier-based device using the method of limits. A temperature of 32°C was set with an increased at a 1°C/s rate, to a maximum of 52°C. The thermode was fixed to the skin on the ventral surface of the mid-forearm. The HPT was defined as the mean of three following measures of painful temperature. The thermode remained on the left ventral forearm; its position was slightly altered between trials to prevent either response suppression of the cutaneous heat nociceptors or sensitization (Schestatsky et al., 2011).

(e) To assess descending pain modulatory by a heterotopic noxious stimulus (CPM), we set the temperature to the point that each patient rating 6/10 pain on the NPS(0-10). The CPM test was performed 30 s following 1-min immersion of the non-dominant hand in cold water (zero to one degree Celsius). The CPM-task was the score in the NPS 0-10 induced by the QST during the cold-water immersion (QST + CPM) at the temperature produced by the QST that they were rating 6/10. CPM-task test was performed after we measured the parameters of cortical excitability. To control for individual variability, the proportion of difference from baseline was used was used for the analysis (Botelho et al., 2016).

(f) Daily sleep quality was measured by a 10-cm visual analog sleep quality scale (VASQS) which scores ranged from zero (the worst sleep quality) to 10 (the best sleep quality). The following question was asked to patients to answer in their sleep diaries: “How well did you sleep last night compared with your habitual sleep?”

(g) The assessment of the cortical excitability parameters, recordings via surface electromyography placed at the contralateral right first dorsal interosseous muscles using Ag/AgCl electrodes were used. Firstly, the Resting Motor Threshold (RMT) was established by obtaining five MEPs with the peak-to-peak amplitude of at least 50 μV out of ten successive trials. Afterward, we documented ten MEPs with an intensity of 130% of the individual RMT. Additionally, we determined the CSPs during muscle activity measured by a dynamometer at 20% of the maximal force. Ten CSPs were assessed using an intensity of 130% of the RMT. An inter-stimulus interval of 2 ms was used to evaluate the SICI. We set the conditioning (first) stimulus at 80% of the RMT, and we set the test stimulus at 100% of the individual MEP magnitude. We determined the ICF with an inter-stimulus interval of 12 ms. We measured the paired-pulse of TMS in a randomized arrangement for a total of 30 trials (ten for each control stimuli, ICF, and SICI). Off-line analyzes included the collection of the amplitudes of all of the MEPs, SICI and ICF as well as the extension of the CSPs. The equivalent units for these parameters included MEP in μV, ICF and SICI in their ratio to the MEP, and ms for the CSP (Pascual-Leone et al., 1994).

##### Other instruments and assessment

All psychological instruments used in this study had been validated for the Brazilian Portuguese (Caumo et al., 2013; Sehn et al., 2012). Two independent medical investigators blinded to the treatment group assignments were trained to apply the pain scales and to conduct the psychological tests. The patients’ baseline depressive symptoms were estimated using the Beck Depression Inventory II (Wang and Gorenstein, 2013). The sleep quality was determined using the Pittsburgh Sleep Quality Index (Bertolazi et al., 2011). Anxiety was assessed using the refined version of the Rash analysis of the State-Trait Anxiety Inventory (STAI) (Kaipper et al., 2010). The pain-related catastrophic thinking was evaluated using the Brazilian Portuguese Catastrophizing Scale (BP-PCS) (Sehn et al., 2012). Demographic data and medical comorbidities were determined using a standardized questionnaire. At the baseline and the end treatment, blood samples were collected to measure to serum BDNF. These blood samples were centrifuged at 4500 × *g* in plastic tubes for 10 min at 4°C and were stored at -80°C for hormone assay. An Enzyme-Linked Immunosorbent Assay (ELISA) using a ChemiKine BDNF Sandwich ELISA Kit, CYT306 (Chemicon/Millipore, Billerica, MA, United States) was used to determine the serum BDNF. The lower detection limit of the kit is 7.8 pg/mL for BDNF.

### Statistical Analysis

**FIGURE 3:**
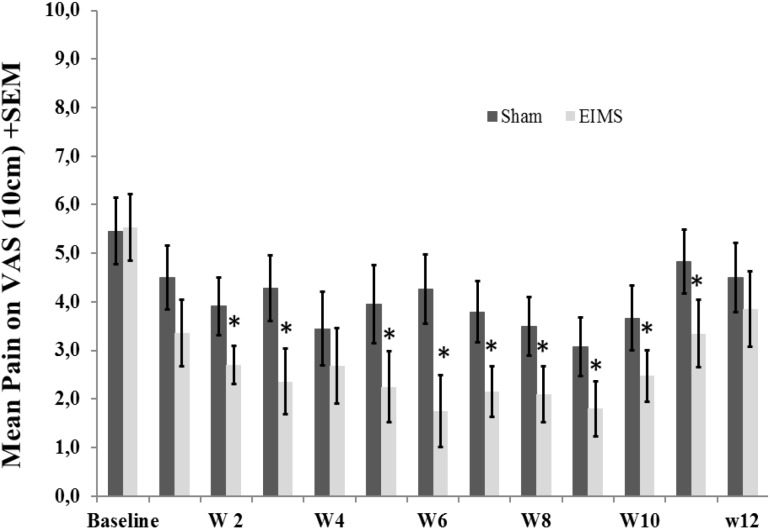
Weekly mean pain levels (as assessed by VAS) from baseline week (W) zero to W12 in the two experimental groups for the following question: “considering your chronic pain that motivated the treatment – how intense was your worst pain during the last 24 h?”. The error bars indicate the standard error of the mean (SEM). Asterisks (^∗^) positioned above the bars indicate significant differences (*P* < 0.01) at those time points between the sham and the EIMS groups. All comparisons were performed by a mixed analysis of variance (ANOVA) model, followed by the Bonferroni correction for *post hoc* multiple comparisons.

**FIGURE 4:**
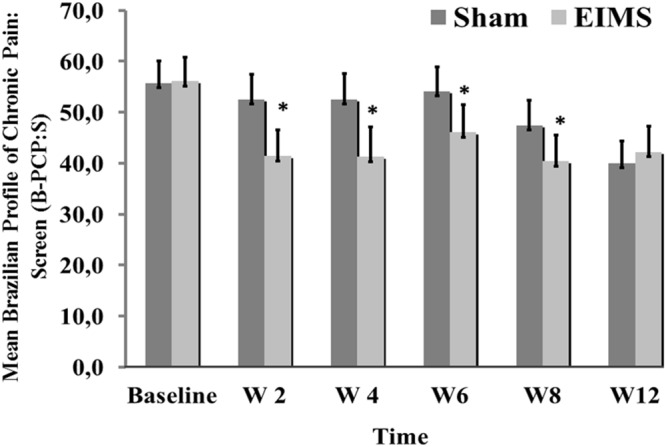
Weekly mean pain and disability related to pain (as assessed by B-PCP:S) from baseline week (W2, W4, W6, W8, and W12) in the two experimental groups. The error bars indicate the standard error of the mean (SEM). Asterisks (^∗^) positioned above the bars indicate significant differences (P < 0.01) at those time points between the sham and the EIMS groups. All comparisons were performed by an analysis of Variance in the Mixed Model, followed by the Bonferroni correction for post hoc multiple comparisons.

*T*-Tests for independent samples and Chi-squared or Fisher’s exact tests were used to compare continuous and categorical variables between intervention groups, respectively. The analysis of the effect of the interventions on the outcomes (VAS for pain scores, B-PCP:S scores, daily sleep quality and changes on NPS(0-10) during CPM-task) was determined using a mixed ANOVA model. The independent variables were time, experimental group (EIMS or sham), the interaction between time and experimental group, and the subject identification as a within-subject factor. (**Table 2** and **Figures 3**, **4**).

To evaluate the mean difference in the cortical excitability measures (MEP, SICI, ICF, CSP) we calculate the percentage change at baseline to end treatment, which were compared using the Wilcoxon–Mann Whitney. The cortical excitability measures at the end of treatment we lost in one patient of EIMS group. Thus, to carry forward analysis using the intention-to-treat approach, we considered the effect observed in the worst case of the respective treatment group (**Table 3**). To assess if the neurophysiological state of the corticospinal pathway and the neuroplastic changes associated with the treatment, we analyze the effect of group in a mixed regression model (**Table 4**). The dependent variable was the cumulative mean B-PCP:S score from baseline to end follow up.

The covariates were the change in the average of serum BDNF expressed in percentage and the baseline MEP. All analysis were adjusted for multiple comparisons by the Bonferroni’s test. The data were analyzed using SPSS version 22.0 (SPSS, Chicago, IL, United States).

## Results

### Patients Characteristics

Patients demographic and clinical features are shown in **Table 1**. Twelve patients were assigned to the sham group, and 12 were allocated to the EIMS group. Twenty-three patients completed the study; one patient in the EIMS group withdrew due to needle phobia. The baseline characteristics were similar across the EIMS and sham groups (all *P*-values > 0.05) (**Table 1**). We did not observe any severe or moderate side effects from the interventions.

**Table 1:** Characteristics of the study sample.

	Placebo-sham (*n* = 12)	EIMS (*n* = 12)	*P-*value^∗^
Age (years)	46.00 (13.55)	48.36 (10.97)	0.66
Education (years)	13.40 (3.48)	12.18 (3.60)	0.44
Smoking (Yes)	2 (16.67%)	1 (8.33%)	0.51
Clinical Comorbidity (Yes)	7 (58.33%)	9 (75%)	0.3
Hypertension	4 (33.33%)	2 (16.67%)
Hypothyroidism	1 (8.33%)	—
Other	2 (16.67%)	3 (25%)
History of psychiatric disease (Yes)	6 (50%)	7 (58.33%)	0.15
Global pain on visual analogue scale	5.89 (3.20)	5.70 (3.49)	0.9
Pittsburgh Sleep Questionnaire	17.6 (±7.6)	19.0 (±5.9)	
Beck Depression Inventory	14.40 (8.63)	16.82 (10.90)	0.31
State-Anxiety on STAI	23.80 (18.35)	22.82 (7.47)	0.74
Trait-A anxiety on STAI	26.80 (8.35)	27.73 (8.93)	0.92
Brazilian Portuguese Catastrophizing Scale (B-PCS)	29.00 (15.43)	28.45 (11.86)	0.93
Profile of Chronic Pain: Screen for a Brazilian population (B-PCP:S)	60.58 (14.96)	60.78 (11.39)	0.88
Pain intensity reported on B-PCP:S	24.75 (3.05)	23.65 (3.80)	0.47
Interference with activities reported on B-PCP:S	19.08 (7.26)	21.70 (8.37)	0.44
Emotional burden due pain reported on B-PCP:S	16.10 (7.61)	16.08 (5.97)	0.82


*Values are given as the mean (SD) or frequency (*n* = 24).*

### Primary Outcomes: Efficacy Concerning Pain Scores and Disability

#### Pain Scores on VAS

After treatment, the EIMS group had significantly lower pain scores in the VAS (*P* < 0.001) than the sham group (**Table 2**), and there was no interaction between time and intervention group (*P* = 0.92) (**Figure 2**). Compared to the sham group, the EIMS group displayed a relative mean pain reduction of -73.02% (effect size of 0.55) at 12 weeks after the conclusion of the interventions (**Table 2**). Compared to baseline, the superior effect of EIMS was also evident: in the second week of treatment the EIMS group had a pain reduction of 55.31% compared to baseline.

**Table 2:** Treatment effect on pain, sleep quality, cortical excitability parameters, and descendent modulator system between Groups: Mean ± SD, percentage on mean change before (B) to after (A) treatment, mean difference with the confidence interval (95% CI) and effect size (CI) (*n* = 24).

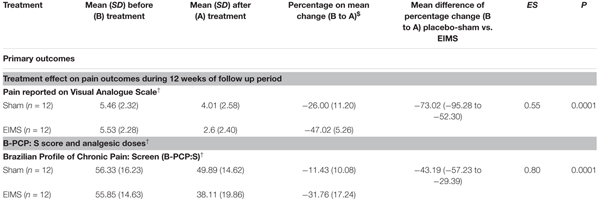

*^$^Mean difference in the on mean change before (B) to after (A) between treatment groups (rTMS vs. placebo-sham). ^†^Mixed ANOVA model. Mean difference groups. Compared using Wilcoxon–Mann Whitney. Effect size (ES) (Mean difference EIMS vs. Placebo-sham)/Standard deviation on placebo-sham] 12 weeks after conclude the treatment. The effect size was defined as small if lower than 0.20; moderate if between 0.50–0.60; and large if larger than 0.80.*

#### Disability Due to Pain Assessed by the B-PCP: S Score

The EIMS group had significantly higher improvement in the mean B-PCP:S score (*P* < 0.000,1) (**Table 2**). There was effect of time and interaction between time and intervention group (*P* = 0 < 001, for both). The changes on B-PCP:S in follow-up was presented in **Figure 4**. This effect persisted until the 8th week following the end of the intervention, with an effect size of 0.8.

### Secondary Outcomes

#### Use of Analgesics, Conditioned Pain Modulation, Neurophysiological Changes and Sleep Quality

##### Use of analgesics

Analgesic use was reported in 69.4% during the treatment period in the sham group and 30.6% in the EIMS group. The relative risk for using analgesic during the 14 weeks (two treatment weeks and 12 weeks of follow-up) was 2.95 (95% CI, 1.36 to 6.30); that is, the sham group used almost threefold additional analgesics. There was a significant decrease in the analgesic doses for patients receiving EIMS compared to those receiving sham (*P* < 0.01).

##### Effects on HPT and conditional pain modulation by heterotopic stimulus

**FIGURE 5:**
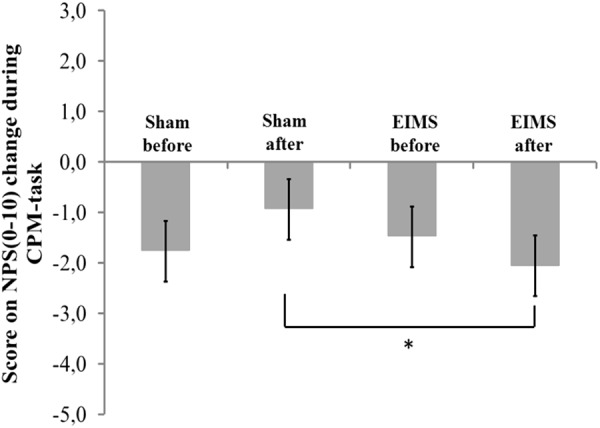
The change in NPS (0–10) during CPM-task, at baseline before intervention and in the end of treatment in the two experimental groups. The error bars indicate standard error of the mean. Asterisk (^∗^) indicates differences between the sham and EIMS groups. All comparisons were performed by a mixed analysis of variance model, followed by the Bonferroni test for *post hoc* multiple comparisons. Numerical Pain Scale (NPS0–10).

The EIMS increased the HPT (**Table 2**) and induced a 61.27% reduction in the pain scores on the NRS during the evoked pain by QST vs. QST + CPM (**Figure 5**). These findings suggest that the EIMS-intervention also induced an effect on the bottom up inhibitory mechanisms.

The scores on numerical pain rating scale before treatment (QST vs. QST + CPM) in EIMS and sham group was -1.49 (1.99) vs. -1.77 (3.09) (*P* > 0.05), respectively. After ten sessions of treatment, the change on the NPS(0-10) during CPM-task in EIMS and sham was -2.04 (0.79) vs. -0.94 (1.18), respectively. The difference mean was – 1.25 (-2.07 to -0.18), (*P* = 0.01).

##### Neurophysiological changes: assessment of EIMS effect on cortical excitability – indexed in TMS parameters

Compared to the group allocated to sham, the EIMS decreased the MEP by 28.79% (**Table 3**) (*P* = 0.02) and increased the SICI by 37.41% (*P* = 0.005). However, the EIMS did not induce significant changes in ICF and CSP (**Table 2**).

**Table 3:** Treatment effect on sleep quality and cortical excitability parameters between Groups: Mean ± SD, percentage on mean change before (B) to after (A) treatment, mean difference with the confidence interval (95% CI) (*n* = 24).

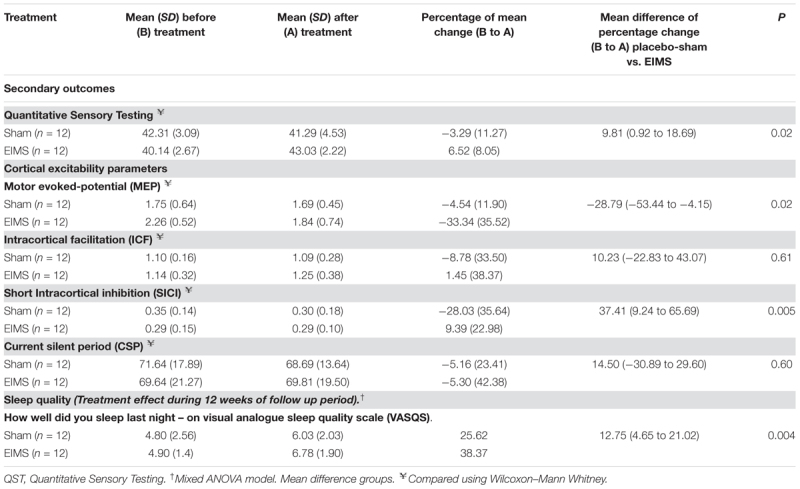

##### Assessment of sleep quality

There was no interaction between time and intervention group for the previous night sleep quality compared with the habitual sleep quality based on the VAS-QS scores (*P* = 0.004). However, the EIMS improved the VAS-QS by 12.75% in the previous night sleep quality compared with habitual sleep.

##### Neuroplasticity markers that predict a long-term impact of EIMS on disability due to pain

One crucial issue is to identify markers associated with the long-term effect on pain and disability assessed by B-PCP:S. To address this critical matter, we run a mixed regression model in which we controlled the change on the B-PCP:S score from the end of treatment for both parameters, MEP at baseline and the change on serum BDNF from baseline to end treatment (**Table 4**).

**Table 4:** Markers that predict the long term effect of treatment on pain and disability assessed in a multivariate mixed regression model (*n* = 24).

Dependent variable: Brazilian Profile of Chronic Pain: Screen (B-PCP:S)				

Parameter	β	*SEM*	*t*	*P*	*95% CI*
Intercept	89.14	8.487	10.503	0.000	72.33 to 105.94
*Treatment group*					
EIMS	-9.89	2.865	-3.454	0.001	(-15.57 to -4.22)
Sham	0^(reference)^				
Motor evoked potential (MEP) at baseline	-0.61	0.153	-4.03	0.000	(-0.58 to -0.26)
(BDNF)^&^	-0.08	0.132	-0.70	0.48	(-0.33 to 0.16)
*Interaction between Change on serum brain derivate neural factor (BDNF)^&^ vs. treatment group*			
EIMS ^∗^ BDNF^&^	0.67	0.29	2.24	0.02	(0.07 to 1.26)
Sham ^∗^ BDNF^&^	0^(*reference*)^				


*CI, confidence interval; EIMS, electrical intramuscular stimulation. ^&^Change on serum brain-derived-neurotrophic factor (BDNF) [(BDNF pre-treatment – BDNF after treatment)/BDNF pre-treatment]^∗^100.*

At the baseline, the serum BDNF in sham and EIMS groups was 27.11 (10.97) vs. 26.21 (6.83), respectively. While at end treatment the serum BDNF in the sham and EIMS groups was 25.05 (10.45) vs. 31.93 (10.74), respectively. Thus, to adjust to individual characteristics, we calculated the percentage of change from baseline to treatment end. The mean (SD) of the percentage of change in the sham and EIMS groups was -6.06 (19.53) vs. 28.74 (25.57), respectively. The Mann–Whitney Test showed that sham group had a significantly lower serum BDNF (*P* < 0.01) compared to EIMS when we compared the mean of percentage change from the baseline. Afterward, we ran a multivariate mixed regression model, which showed an interaction between treatment groups (EIMS or sham) and BDNF (*P* < 0.05) (**Table 4**). This result suggests that the increase in the BDNF is intervention dependent. Also, it indicates that the change in BDNF induced by EIMS may be a marker that predicts the long-term effect of treatment, as assessed by the B-PCP:S and the VAS 12 weeks after end treatment. Our findings showed that more substantial increase in serum BDNF induced by the EIMS and that higher excitability in the corticospinal pathway as measured by the MEP at baseline, both were correlated negatively with pain and disability at the end of follow-up. Also, it indicates that EIMS effect induced the more significant increase of serum BDNF.

## Discussion

This study demonstrated that EIMS induced a sustained improvement in pain and disability, as well an increase in the inhibition of the corticospinal system as indexed by the MEP amplitude. The effects of EIMS improved the HPT, the sleep quality, and the DPMS function. Additionally, we observed that the MEP before treatment and the more significant change in the serum BDNF at the treatment end predicted the long-term effect of EIMS in pain and disability at the follow-up end.

These findings extend the knowledge regarding the effects of EIMS on pain and disability according to daily pain scores on VAS, the B-PCP: S score (**Table 2**) and reduced analgesic use at follow-up end. Assessed by different ways, these findings also highlight the long-term clinical impact of the bottom-up effects of EIMS, which is associated with changes in serum BDNF, as well as neurophysiological and psychophysical measures. Overall, this indicates that neuroplastic changes in the pain pathways mediated the clinical result. The impact of EIMS on pain scores is consistent with those of previous randomized clinical trials in which the EIMS using a frequency of 2 Hz induced better effects than sham in a short time in MPS (Couto et al., 2014). Also similar to the results of another study, which showed that ten sessions of EIMS using a frequency of 2 Hz in MPS improved the DPMS according to changes in scores on the NPS(0–10) during CPM-task (da Graca-Tarragó et al., 2015).

The effect of EIMS reduced the MEP amplitude and increased the SICI (**Table 2**). Both indicate a reduction in the excitability of the motor cortex, as well as lower facilitation of the transmission at corticospinal neurons. These results showed that electrical stimulation of peripheral nerves could modulate cells of cortical networks (Miyata and Usuda, 2015). The MEP amplitude reflects the ratios of glutamine/glutamate and GABA/glutamate in the corresponding primary motor cortex. Thereby, larger MEP amplitude indicates hyperactivity in glutamatergic circuits or loss of GABA-ergic activity mediated by GABA-A receptor (Lin et al., 1996; Dall’Agnol et al., 2014). Thus, the current finding highlight that the M1 stimulation permits us to assess if the EIMS effect might reduce the facilitation in the corticospinal pathway. Accordingly, EIMS seems to be able to modify the inhibitory and excitatory interhemispheric interactions to enhance the neuroplasticity and possibly the balance between excitation and inhibition. Even though the mechanisms underlying of the EIMS effect on cortical excitability are unclear, the neuroplasticity process induced by EIMS was likely counter-regulated by the disinhibition state at the cortical level and within the interconnections involved in pain modulation. This finding is plausible because there is evidence that the cortex modulates the nociception by projections directly into the spinal dorsal horn neurons and trigeminal nucleus (Millan, 2002). These circuits can also be mediated by indirect projections from the cortex to the dorsal horn through the hypothalamus, amygdala, and PAG (Millan, 2002) or from the secondary somatosensory cortex through thalamus relay (Xie et al., 2009). In fact, these findings indicate that the modulatory effects produced by EIMS were not limited to the spinal cord but also occur at distant interconnected sites including the motor cortex.

The present data also extend literature the larger MEP at baseline is inversely correlated with pain severity and disability at follow-up end (**Table 3**). This inverse correlation suggests that MEP at baseline indicates higher excitability in a cortico-spinal way, that is part of central sensitization concurs to a higher impairment due to pain. This hypothesis is plausible because, in central sensitization (CS), the secretion of BDNF by astrocytes changes Gamma-aminobutyric acid (GABA-ergic) function, where it can induce excitability rather than inhibition. Indeed, the CS is a dysfunction which involves different mechanisms, such as a selective loss of GABAergic interneurons (Moore et al., 2002) and the collapse of the chloride gradient, which is correlated with enhanced excitability in postsynaptic neurons (Coull et al., 2003). Thus, this result suggests that the adjustment of the imbalance between excitability and inhibition is controlled by different neurobiological systems, and perhaps some processes are influenced by EIMS. As the MEP is an index of the hyperexcitability in corticospinal pathways, it reflects that the motoneuronal excitability at baseline can predict at some level the clinical effect of EIMS at the follow-up end. In fact, our findings may hold critical clinical implications such as (i) to support an understanding of the bidirectional pathways between peripheral and central brain changes in MPS. (ii) To help to decide on a therapeutic approach based on the neurophysiological phase state of each patient, because a larger MEP amplitude at baseline predicted more considerable improvement in the disability due to MPS. (iii) To improve the understanding of underlying neurophysiological mechanisms of the limitation due to chronic MPS, which could give support to plan neuromodulatory approaches to induce a top–down (i.e., direct current stimulation-tDCS) and bottom-up modulation technique (i.e., dry-needling) or pharmacological interventions.

Also, we observed that the EIMS effect increased the SICI by the reorganization of the somatosensory cortex. This hypothesis is acceptable because a similar effect has been demonstrated using electroacupuncture in neuropathic pain (Napadow et al., 2007). Also, electroacupuncture leads to a reduction in inhibitory postsynaptic currents mediated by GABA (Fu and Longhurst, 2009), through a process that is aided by BDNF released from microglia. Accordingly, the electroacupuncture can induce long-term depression (LTD) (Pyne and Shenker, 2008), which is an activity-dependent reduction in the efficacy of neuronal synapses that modify the expression of the postsynaptic receptor NMDA (*N*-methyl-D-aspartic acid) (Pérez-Otaño and Ehlers, 2005). Thereby, EIMS may modulate the disinhibition process due to the loss of the postsynaptic potassium chloride cotransporter (KCC2).

This result is clinically relevant and suggests that the increase in serum BDNF levels underlie the therapeutic effect of EIMS. This relationship between the increase in BDNF and pain is consistent with evidence provided by a study in patients with chronic tensional headache treated with electroacupuncture (Chassot et al., 2015). Another study patients with MPS found a similar effect in patients undergone treatment using repetitive (r)TMS sessions (Dall’Agnol et al., 2014). Thereby, these findings give additional neurobiological support for the sustained impact of EIMS on pain, and it suggests that the serum BDNF may be a useful marker to predict its therapeutic effects at long-term.

The EIMS effect increased the HPT and improved the DPMS as evidenced by the changes in the NPS(0–10) during CPM-task. These findings suggest that the EIMS-intervention induces an effect on the bottom–up inhibitory mechanisms. Its impact in the HPT is in agreement with an earlier study, in which EIMS stimulation of myofascial trigger points (MTP) produced an increase in pain threshold and greater activation on the dorsal midbrain encompassing periaqueductal gray (PAG) (Niddam et al., 2007). In our findings, we demonstrated that EIMS induced enhancement on descending pain control mechanisms (**Figure 5**). Accordingly, it is plausible that it activates the PAG, which is the primary control center for descending pain modulation (Pertovaaraa and Almeida, 2006). Further, our findings showed that EIMS improved pain and disability, and these changes were concurrent with the improvement in the neurophysiological parameters (e.g., SICI, MEP and in DPMS [change in the NPS(0–10) during CPM-task]). In fact, these effects are congruent with the idea that the descending pain facilitation is a process mediated by structural plasticity. These changes occur in an activity-dependent manner in the pain pathways, an effect mediated by serotonin, glycine, or GABA neurotransmitters, which likely engage connectivity forces at cortical and infra-cortical levels (Basbaum and Fields, 1978; Cui et al., 1999).

The EIMS effect also improved the restorative effect of sleep (**Table 2**) as in agreement with a previous study the EIMS improved sleep quality in a short-term (Couto et al., 2014). In fact, in the present study, the EIMS effect on sleep quality presented a small effect size. However, it is possible that EIMS changes the sleep quality by indirect effects such as modulation of the sleep/wake cycle secondary to an increase of melatonin secretion (Spence et al., 2004). This benefit may be a secondary effect of the needling procedure, as it could reduce the levels of circulating cytokines as shown in the experimental pain neuropathic model, where its effect decreased the inflammatory mediators (Cha et al., 2012). Thus, according to another evidence of a clinical study, the increase of cytokines could interrupt melatonin secretion by the pineal gland (Spence et al., 2004). Although, this a possible hypothesis to explain this association and further studies are needed before definitive conclusions are drawn.

Some issues concerning the design of our study must be addressed. (i) Our sample comprises only female because exist extensive literature that they are more prone to activation upon negative emotional responses (i.e., stress, fear, and anxiety) as well to physiological factors (i.e., the capability to endure pain) (Keefe et al., 2001; Wiesenfeld-Hallin, 2005). If for one side the homogeneity of this study population is methodologically advantageous to reduce the effect of potential confounding factors, and thus, permit us to understand the impact of EIMS in the corticospinal pain modulation system, it has the disadvantage to restricts the generalizability of results. Therefore, other researches with a higher number of patients are needed to more widely assess the potential benefits of EIMS in different clinical settings. Hence, this could give support to therapeutic decision making in clinical settings. (ii) Even though we have included only patients without prior contact with acupuncture, the acupuncturist-physician knew the type of intervention that was applied. Also, EIMS produces a muscle movement while sham stimulation not. Hence, the sensory perception could increase the chance of patients guess the type of intervention. However, we must realize that these limitations are intrinsic to technique. (iii) Given we did not formally measure the awareness of the allocation group (either active or sham), this is a limitation that one could consider. Despite these limitations, our outcomes were correlated with neurophysiological and psychophysical parameters, which are measurements less prone to bias. Furthermore, we measured the impact of EIMS on pain and disability in the long term; this attenuates the likely impact that this bias could have in our conclusions. The strengths of the study include our assessment that EIMS has a direct effect on the neuroplasticity process using markers of neuroplasticity such as BDNF. At the same time, we evaluated its impact on the clinical outcomes related to pain and disability with a follow-up according to the recommendations of the IMMPACT (Dworkin et al., 2005). Thereby, this study represents a significant contribution to evidence-based therapeutics for EIMS in the treatment of MPS.

## Conclusion

In conclusion, these results revealed that ten sessions of EIMS reduced pain score and improved disability due to chronic MPS, as well reduced the analgesic use. They also suggested that the EIMS effects on chronic MPS are mediated by bottom–up regulation mechanisms, enhancing corticospinal inhibition and that this effect involves an increase in BDNF secretion. Additionally, they suggest that the MEP amplitude before treatment and the changes induced by the EIMS in the serum BDNF predicted it’s the long-term impact on the chronic MPS symptoms.

## Author Contributions

IT, FF, and LB were responsible for maintaining the study records. LB and IT participated in the sequence alignment and drafted the manuscript. LB administered the interventions. LA, AD, and AB realized the outcome measures. MZ participated in the sequence alignment. FF participated in the design of the study and performed the statistical analysis. IT and WC conceived the study, participated in its design and coordination, and helped in drafting the manuscript.

## Conflict of Interest Statement

The authors declare that the research was conducted in the absence of any commercial or financial relationships that could be construed as a potential conflict of interest.
